# Longitudinal monitoring of cancer cell subpopulations in monolayers, 3D spheroids, and xenografts using the photoconvertible dye DiR

**DOI:** 10.1038/s41598-019-42165-2

**Published:** 2019-04-05

**Authors:** Sam Osseiran, Lauren A. Austin, Taylor M. Cannon, Chuan Yan, David M. Langenau, Conor L. Evans

**Affiliations:** 1 Wellman Center for Photomedicine, Harvard Medical School, Massachusetts General Hospital, 149 13th Street, Charlestown, Massachusetts 02129 USA; 2 Harvard-MIT Division of Health Sciences and Technology, 77 Massachusetts Avenue E25-518, Cambridge, Massachusetts 02139 USA; 3 Ludwig Center at Harvard, Harvard Medical School, 200 Longwood Avenue, Boston, Massachusetts 02115 USA; 4 Molecular Pathology Unit, Massachusetts General Hospital, 149 13th Street, Charlestown, Massachusetts 02129 USA; 5 Center for Cancer Research, Massachusetts General Hospital, 149 13th Street, Charlestown, Massachusetts 02129 USA

## Abstract

A central challenge in cancer biology is the identification, longitudinal tracking, and -omics analysis of specific cells *in vivo*. To this aim, photoconvertible fluorescent dyes are reporters that are characterized by a set of excitation and emission spectra that can be predictably altered, resulting in a distinct optical signature following irradiation with a specific light source. One such dye, DiR, is an infrared fluorescent membrane probe that can irreversibly undergo such a switch. Here, we demonstrate a method using DiR for the spatiotemporal labeling of specific cells in the context of cancer cell monolayer cultures, 3D tumor spheroids, and *in vivo* melanoma xenograft models to monitor the proliferation of cellular subpopulations of interest over time. Importantly, the photoconversion process is performed *in situ*, supporting the pursuit of novel avenues of research in molecular pathology.

## Introduction

Fluorescent labeling strategies are widely used in microscopy applications in order to glean a proper understanding of the biomolecular mechanisms underlying normal and aberrant cellular behaviors^1–3^. These techniques are highly diverse: standard immunofluorescence (IF) can be used to tag proteins of interest with high specificity^4^; genetic engineering can allow for the genomic insertion of fluorescent reporter proteins to monitor expression of particular genes^5–9^; organelle-specific fluorescent dyes can also be used for quantitative and/or qualitative cytological measurements^10–12^. Such methods have been used to study key microscopic processes in models spanning monolayers and 3D spheroids *in vitro*^2,10^ all the way up to animal models and humans *in vivo*^13–18^. However, despite their widespread adoption in microscopy, traditional fluorescence-based techniques are static in their labeling. In other words, they cannot be used to monitor specific cell populations of interest that have been identified after the initial labeling step. For example, individual cells within a population of fluorescently labeled stem cells cultured *in vitro* or injected in an animal xenograft model cannot be monitored longitudinally to study their differentiation, as the dye’s fluorescence is an intrinsic molecular property and is therefore independent of the cell’s phenotypic behavior^19,20^.

In fact, there exists a wide variety of applications where the monitoring of specific cell subpopulations over a particular time course would be of great biological relevance. Biomolecular reporters such as green fluorescent protein (GFP), for instance, can be useful in determining whether the cells in a cultured population express a particular gene^7,8^. However, tracking the fate of specific expressing or non-expressing cells within the same culture over prolonged periods would pose a significant challenge^19^. This difficulty is of particular importance in the study of treatment resistance in the context of cancer. In an ideal scenario, cells of interest would first be identified based on the expression of some reporter protein. Those same cells would then be monitored over time as they are challenged with therapeutic strategies in order to ascertain the response to therapy and probe aspects of a cell population’s heterogeneity^21^. One can also envision a similar challenge in the study of immune infiltration within cancerous lesions. For instance, by using the techniques discussed above, the rate of immune cell turnover in the context of immunosurveillance would be extremely challenging to quantify as there is no straightforward manner of distinguishing specific immune cells over a longitudinal series of imaging experiments. While the cumulative influx of leukocytes at any given time can be measured in a fairly straightforward manner using standard fluorescent labeling, the dynamics and rates of immune cell influx and efflux from the monitored area would remain difficult to ascertain due to the inability to distinguish counted from new, yet-uncounted cells^19^. In a similar scenario, cellular metabolic activity can be monitored using chemiluminescence approaches^22^. However, if the goal of the study is to examine and monitor the rate of activation of these cells independently from one another over time, the challenge of distinguishing cells that have already been counted from those that have not once more becomes apparent. These examples highlight some of the current limitations in using conventional fluorescent labeling strategies, particularly in the context of cancer research on the cellular scale.

A great deal of effort has been dedicated to the development of photoconvertible fluorescent labels to fill this biotechnological niche^19,20,23,24^. Much like any other fluorescent molecule, these reporters exhibit a characteristic excitation and emission profile. However, they are distinct from conventional labels in that their optical profile can be predictably and reproducibly converted to a new set of excitation and emission characteristics. The particular set of optical signatures before and after conversion, as well as the reversibility of the process, are intrinsic properties of each photoconvertible reporter. Genetic reporter systems such as Kaede^25^ and Dendra2^26^ have been been found particularly useful^27^, but the natural turnover of fluorescent proteins makes these labeling strategies transient. Many studies could benefit from a photoconvertible approach that has permanence over eight or even ten cell division cycles.

While the excitation and emission properties of fluorescent reporters can range from the ultraviolet all the way to the near-infrared (NIR)^2^, the red end of the spectrum is typically of greatest value for intravital imaging. These longer wavelength signals have a lower tendency to be absorbed and scattered by tissues compared to their bluer counterparts. This allows for increased signal generation and collection, and thus maximizes penetration depth^1,2,28^. One such commercially available NIR fluorescent label known as DiR (1,1′-dioctadecyltetramethyl indotricarbocyanine iodide) is a membrane dye with excitation and emission peaks at 748 nm and 780 nm, respectively^19^. This particular dye has been shown to exhibit irreversible photoconversion upon irradiation with a mere 8 to 45 mW of 750 nm femtosecond pulses over a period as short as 5 to 20 seconds depending on the nature of the sample at hand (i.e. *in vitro* vs. *in vivo*)^19^.

In the context of this study, we sought to demonstrate the use of DiR as a photoconvertible membrane dye for tracking specific cells in solid tissues. These include labeling ovarian cancer cells (OVCAR5) in both 2D monolayer and 3D spheroid cultures, performing photoconversion on a given subpopulation, and monitoring their development over time. The selective isolation of photoconverted cells derived from such *in vitro* cultures using fluorescence-activated cell sorting (FACS) is also demonstrated and highlights the applicability of this method to identify specific cells of interest within a given *in vitro* context. Finally, the photoconversion of DiR is also demonstrated in a melanoma (UACC62) xenograft model in a live zebrafish monitored longitudinally over several days. In building upon prior efforts elucidating the photoconvertible nature of DiR and similar cyanine dyes^19,20,24^, these demonstrations set the stage for novel avenues of research in the context of molecular pathology where longitudinal monitoring of cell lineages can offer insight pertaining to therapeutic response and acquired resistance.

## Results and Discussion

### Photoconversion in Monolayers *In Vitro*

**Figure 1:**
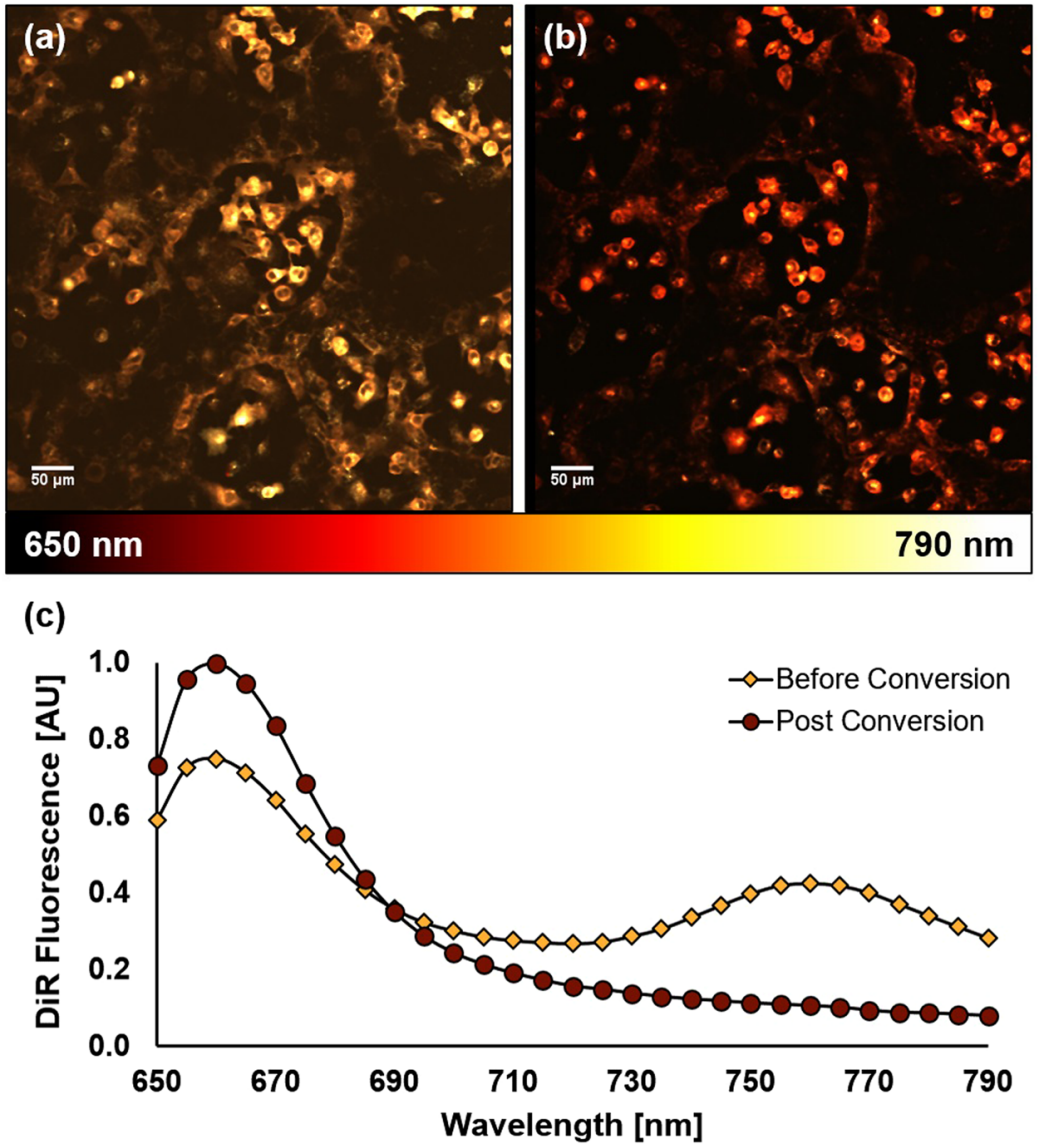
*In vitro* monolayer model of ovarian cancer showcasing the fluorescence of DiR before (**a**) and after (**b**) photoconversion, with associated fluorescence spectra summed across the entire field of view (**c**).

Ovarian cancer cells (OVCAR5) were first grown *in vitro* and allowed to reach confluence before labeling with DiR. Upon illumination with 635 nm laser light, the bright fluorescence signals observed from the stained cells revealed the two characteristic DiR emission peaks located around 660 nm and 760 nm as can be seen in the plotted emission spectra in Fig. 1. By encoding every probed emission wavelength from the scan to a color scale element from Matlab’s “hot” color map, the two standard DiR peaks result in a vivid yellow false color. On the other hand, photoconverted DiR exhibits 2 notable spectral features that distinguish it from its standard counterpart: (1) a marked increase in fluorescence emission at 660 nm, and (2) the elimination of the second peak at 760 nm. This results in a dark red false color, allowing for a simple and intuitive visual distinction between converted and non-converted cells.

**Figure 2:**
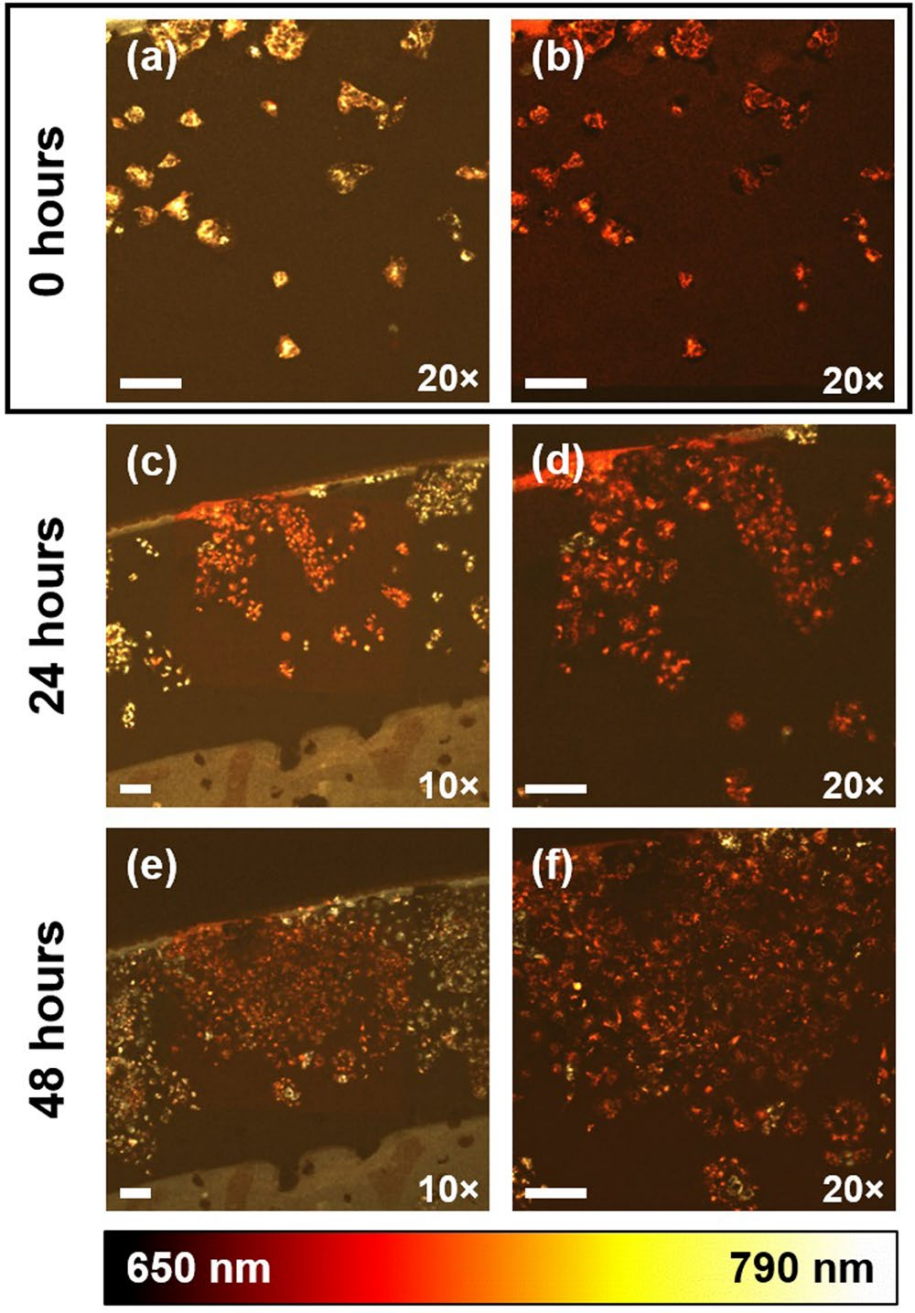
Longitudinal monitoring of an ovarian cancer cell subpopulation of interest, seen before (**a**) and after (**b**) photoconversion on Day 0. The same field of view was revisited 24 hours (**c**,**d**) and 48 hours (**e**,**f**) following the photoconversion process to monitor the cellular proliferation over time. All scale bars correspond to 100 *μ*m. Images in (**a**,**b**,**d**,**f**) and (**c**,**e**) were acquired at 20× and 10× magnification, respectively.

Building upon these results, fresh OVCAR5 cells were newly plated and stained with DiR prior to imaging with 635 nm illumination. A single region of interest (ROI) 636 *μ*m × 636 *μ*m in size (i.e. a full field of view at 20× magnification) was photoconverted on Day 0, where Fig. 2(a,b) show the ROI before and after photoconversion, respectively. The same ROI was then revisited daily over the next 48 hours and imaged at both 10× and 20× magnification (Fig. 2(c–f)). As expected, the cells were observed to be growing normally and retained sufficient amounts of the DiR dye to generate readily detectable fluorescence signals despite multiple cell divisions. Interestingly, after 48 hours following the initial seeding, labeling, and photoconversion, the borders of the ROI became blurred as cells labeled with standard DiR are seen to infiltrate the area and, conversely, cells with photoconverted DiR are observed to expand beyond their site of origin.

Given the permanent spectral shift, this methodology can be widely used in *in vitro* settings where temporal dynamics are of particular scientific interest. For example, cell lines genetically modified with the ability report the expression of a gene of interest using fluorescent proteins can all be uniformly stained with DiR. Then, cells that do express the reporter protein can be photoconverted *in situ* while imaging to permanently mark which individual cells within the heterogeneous population express the gene of interest on Day 0. In this manner, this subpopulation of interest can be monitored within the context of its heterogeneous environment over a time course spanning several days or even weeks as it is challenged with various therapeutic strategies. This methodology would therefore allow one to monitor the genetic state of specific individual cells over time, offering insight pertaining to the expression dynamics of the gene of interest. This methodology can also be applied to cellular co-cultures in order to monitor key interaction parameters of interest between specific members of either cell population.

### Photoconversion of 3D Spheroids *In Vitro* and FACS Capture of Cell Populations

While cellular monolayers cultured *in vitro* are advantageous for study given their ease of use and simplicity, they are not representative of cancer *in vivo*. Indeed, interactions with extracellular matrix elements and neighboring cells in all three spatial dimensions can significantly influence tumor pathophysiology. Three-dimensional *in vitro* tumor culture models can replicate many of the molecular and structural features of human tumors, and therefore offer a convenient middle ground between *in vitro* monolayer cultures and live animal experiments *in vivo*^29^.

There are many situations where a specific subpopulation of cells within a tumor spheroid may be of interest. For instance, one may want to differentiate between the cells located towards the core of a spheroid rather than those on its periphery to study biochemical pathways related to hypoxia in the context of cancer pathogenesis^29–31^. Alternatively, one may expose cultured tumors to particular chemical gradients in order to model the diffusion of therapeutic compounds, and study the resulting tumor response at the cellular population level. In such cases, the ability to visualize, target, and permanently label a portion of the studied tumor would be of significant value, as it would allow one to correlate longitudinal observations with spatially-resolved subpopulations of interest within a tumor throughout its development.

**Figure 3:**
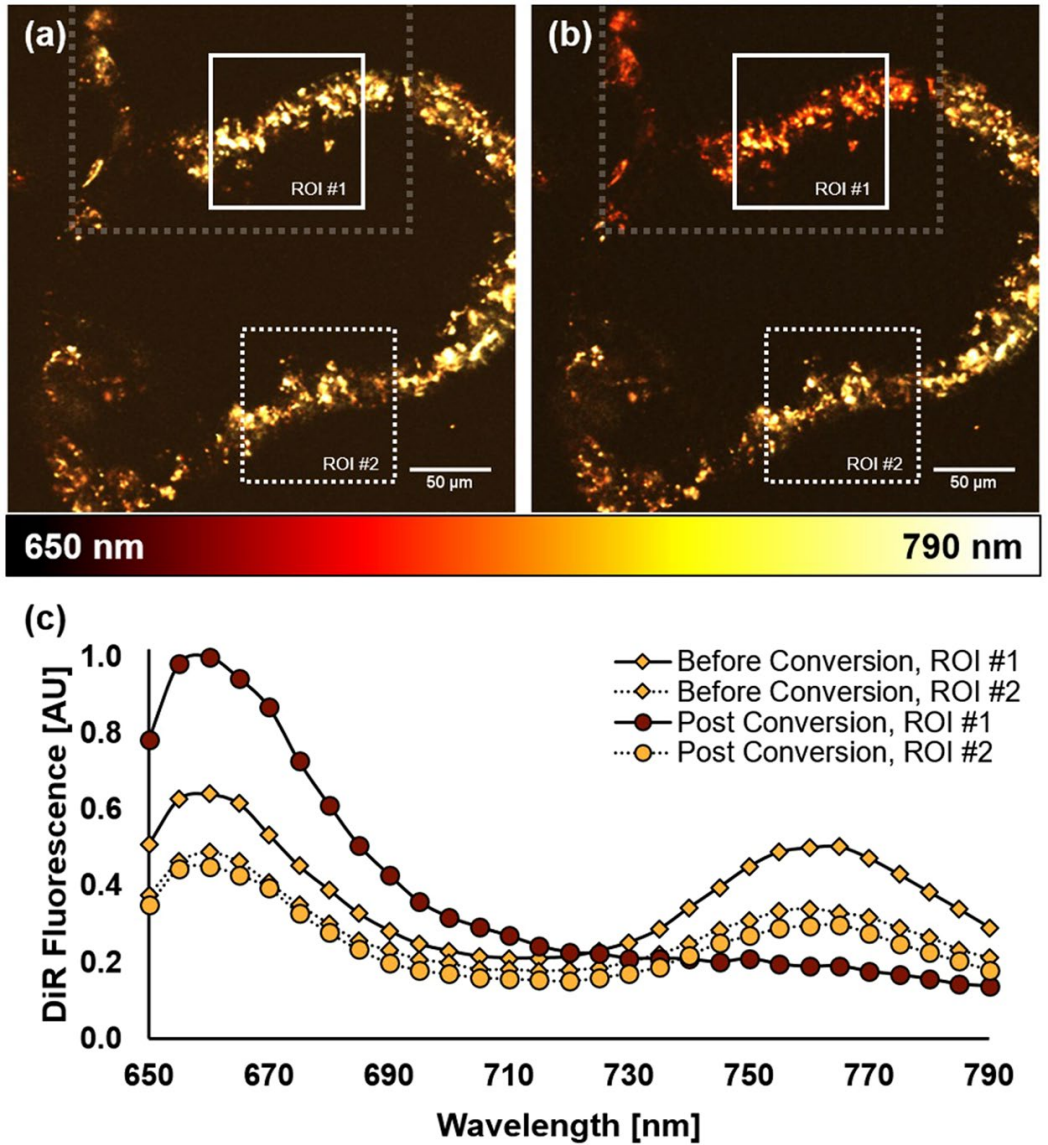
3D tumor spheroid of ovarian cancer cells before (**a**) and after (**b**) photoconversion of the topmost peripheral region indicated by the gray box. Two regions of interest (ROI) have been selected within the image, where ROI #1 encompassed photoconverted cells while ROI #2 did not. The corresponding fluorescence spectra are shown in (**c**), where solid and dashed lines refer to ROIs #1 and #2, and diamonds and circles refer to before and after photoconversion, all respectively.

In order to demonstrate this application, OVCAR5 cells were grown on a bed of Matrigel, a commercially available gelatinous culture substrate that mimics extracellular matrix and favors the formation of 3D spheroids^29,30^. Once these cellular clusters reached a few hundred microns in diameter, a subpopulation of cells along the topmost peripheral section of a tumor spheroid was targeted for photoconversion as seen in Fig. 3. An ROI defined within the targeted area revealed the signature twin peaks of standard DiR at 660 nm and 760 nm prior to photoconversion. Following the conversion process, the second peak was found to disappear while the first peak’s signal increased as expected (Fig. 3(c)). In examining a second ROI defined outside the targeted area, the emission signature of standard DiR was preserved all throughout the procedure. Some minor photobleaching was observed and helps to explain the modest drop in fluorescence signal.

Given a partially photoconverted tumor spheroid where the optical contrast permanently identifies specific cells of interest, it follows that a manner for isolating said cells for subsequent expansion and study would be of great practical value. For example, in the context of ovarian cancer metastasis, spheroids are known to spread intraperitoneally and continue growing at distant sites along the walls of the peritoneum^29^. Here, one may seek to optically label the cancer cells in contact with the endothelial cells along the peritoneal walls to later study their protein expression.

To demonstrate the applicability of this platform for such a scenario, 3D *in vitro* spheroids of OVCAR5 cells were grown across the entire surface of a glass-bottom dish filled with Matrigel-rich media and cultured over the course of 2 weeks. This favored the formation of hundreds of spheroids that are each comprised of many hundreds more cells^29,30^. After registering the location of each spheroid via visual microscopy inspection, the photoconversion routine was automated. At each location, the external pulsed 750 nm laser light was used to illuminate a coarsely-defined z-stack through approximately one half of the spheroids. They were then disaggregated and prepared for cell sorting via FACS.

**Figure 4:**
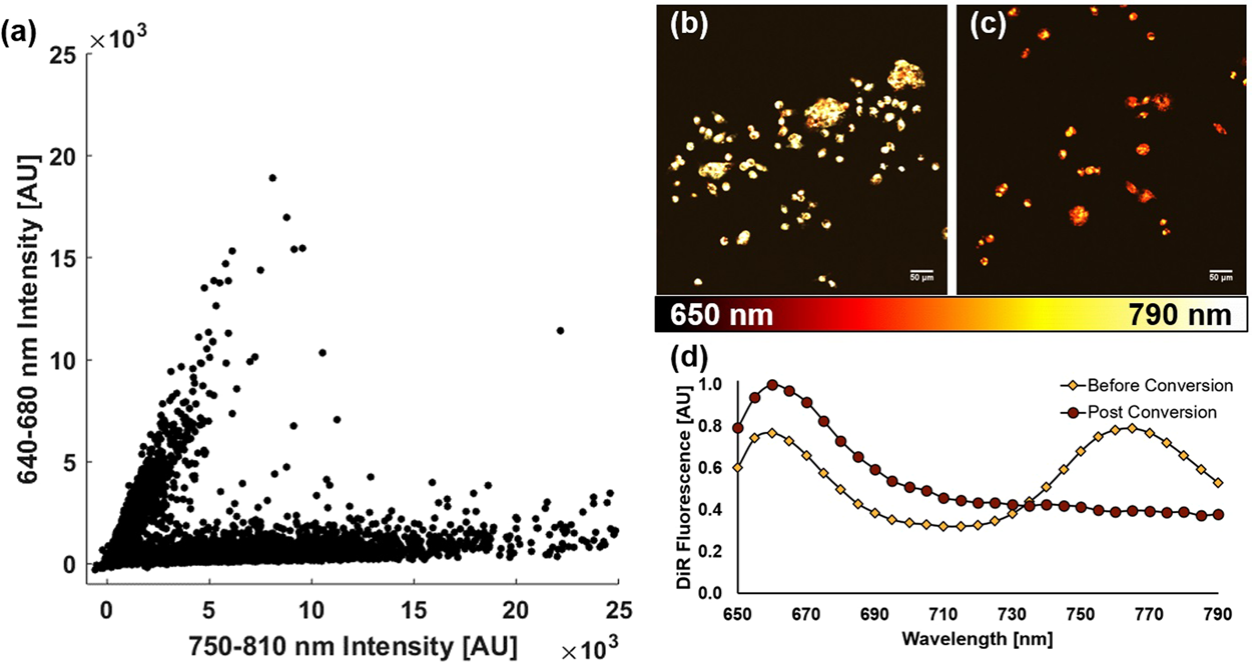
FACS plot of a cell sample from disaggregated, partially photoconverted ovarian cancer spheroids (**a**), and subsequent seeding of sorted non-converted (**b**) and photoconverted (**c**) cells. The fluorescence spectra from the sorted cells are shown in (**d**), demonstrating the irreversible nature of *in situ* DiR photoconversion.

The fluorescence captured from a representative sampling of the disaggregated tumor cells revealed two distinct populations of labeled cells as shown in Fig. 4(a). The fluorescence intensity from the NIR emission band of DiR is shown on the horizontal axis, while the vertical axis indicates red fluorescence, which is markedly increased upon photoconversion. Following the sorting process via FACS, the isolated non-converted and photoconverted cells were plated and cultured as shown in Fig. 4(b,c). These results show that not only was the DiR label retained in the cell membrane despite the multiple processing and handling steps, but both its standard and photoconverted forms were present at readily detectable levels. Moreover, the spectral shift following the photoconversion process was indeed found to be permanent and irreversible, as verified in Fig. 4.

This methodology is therefore particularly applicable in contexts where an especially small subpopulation of cells are of interest for subsequent experimentation. Using *in situ* photoconversion, these cells can be isolated via FACS and then expanded *in vitro* to generate large numbers of cells from the subpopulation of interest.

### Photoconversion in Zebrafish Xenograft Model *In Vivo*

Finally, in the context of *in vivo* imaging, there are many scenarios where the labeling of one cellular subpopulation of interest may be of use. One such example is that of lineage tracing in the context of metastasis: given an initially heterogeneous population of cells within the primary tumor, there is a persistent and challenging need to identify which ones are the first to spread; which ones divide most rapidly; and which cell subpopulations grow and expand despite therapeutic administration.

**Figure 5:**
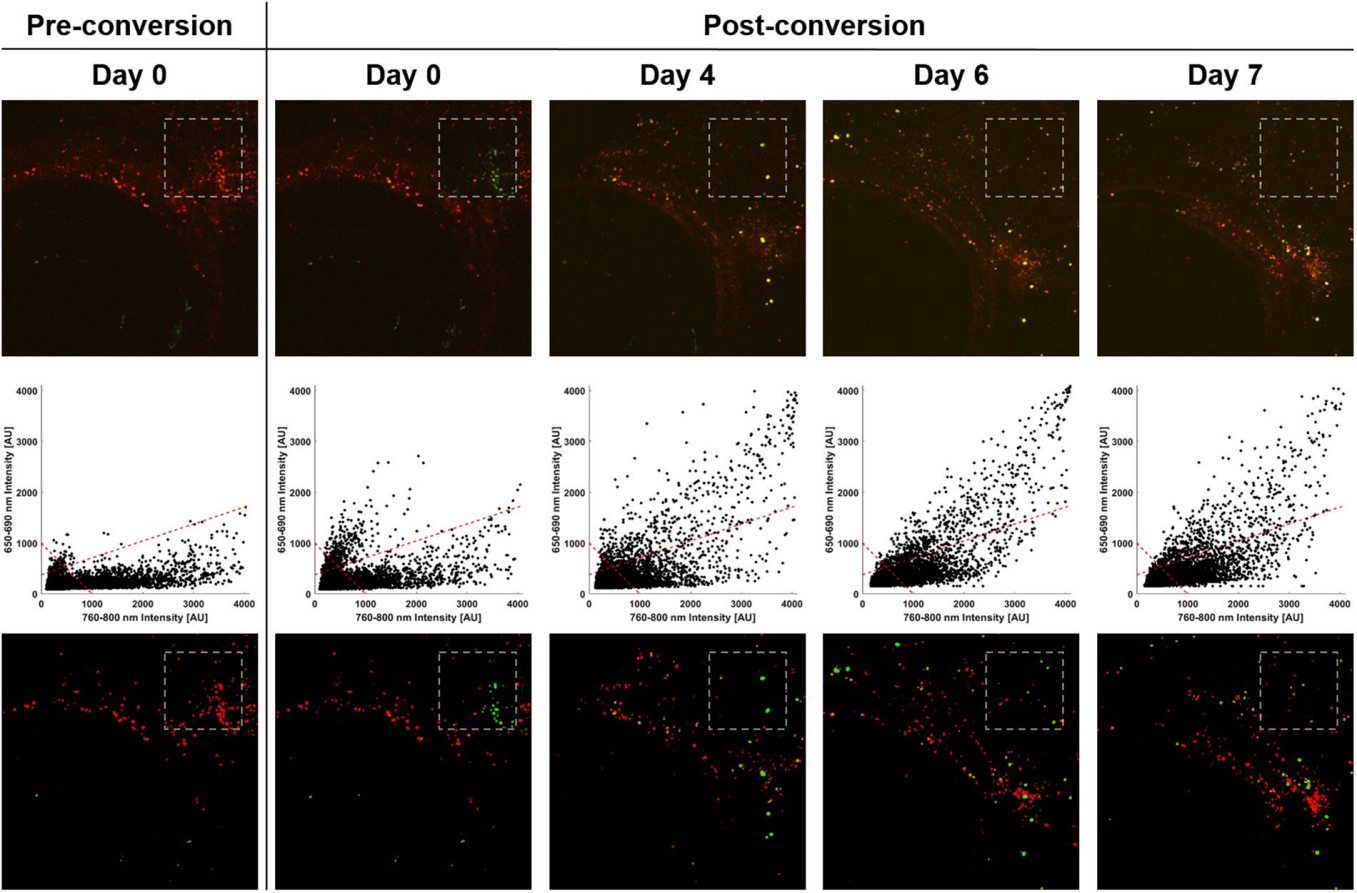
*In vivo* longitudinal monitoring of a photoconverted subpopulation of melanoma cells indicated by the gray box in a zebrafish xenograft model following *in situ* photoconversion. In all images, the eye is located at the bottom-left of the field of view and was consistently used as a fiducial marker throughout the experimental time course to ensure monitoring of the same area over time. The top row shows a colored overlay of the two collected fluorescence emission bands, namely 650–690 nm in green and 760–800 nm in red. The middle row shows a scatter plot of each pixel, where green and red channel intensities are plotted against one another in order to generate a representation visually reminiscent of flow cytometry plots. The red dashed lines in the scatter plots correspond to the gating criteria to identify pixels containing either photoconverted or standard DiR. These gates are then used to recolor the images from the top row, where the images in the bottom row show photoconverted and standard DiR fluorescence in green and red, respectively.

In order to better model such a scenario, melanoma cells were first cultured and labeled with DiR. They were then injected retro-orbitally in zebrafish to produce melanoma xenograft models *in vivo*. After 7 days, the fish were anesthetized and imaged using confocal fluorescence microscopy with 635 nm illumination to visualize the implanted DiR-labeled melanoma cells behind the eye as seen in the leftmost column of Fig. 5 (top panel). A small field of view roughly 364 *μ*m × 364 *μ*m in size containing several tens of cells was then arbitrarily selected for photoconversion. The overlay in the second column of Fig. 5 shows the 760–800 nm intensity in red, while the 650–690 nm signal is shown in green.

The added convenience of such a platform is that images can be processed in a manner similar to flow cytometric analysis, where pixel intensities can be shown in a scatter plot that visually relates one channel’s intensity to that of the other. This familiar data representation then allows for a straightforward gating of the fluorescence signals to identify cellular subpopulations of interest over time in a wide variety of platforms ranging from *in vitro* to *in vivo*. Indeed, as can be appreciated in the bottommost row of Fig. 5, the initial cluster of cells within the photoconverted ROI is clearly visualized on Day 0 and is then seen spreading over the course of the following week, reaching areas far beyond the original photoconversion site. This demonstration is of particular value in the context of *in vivo* animal models of metastasis, where one may photoconvert cells at the primary tumor site that are more or less likely to metastasize. As tumors then form at distant anatomical sites, their spectral signatures would allow one to infer specifically which cells from the heterogeneous primary tumor were the ones to metastasize. The major advantage of this approach, in particular for patient xenograft studies, is that this photoconversion method does not require cellular genomic manipulation as is the case with fluorescent reporters. Cells never have to be transformed, grown on hard plastic surfaces, or exposed to cell culture conditions to prepare for photolabeling. This has the advantage of preserving tumor cellular heterogeneity and may allow for photoconversion techniques to be used with primary samples and minced tumor implantation models^32^. For example, commercially available oils and pastes containing cyanine dyes have been shown to effectively stain cell populations of interest *in vivo*, as in the case of olfactory neuron staining in live zebrafish larvae^33^. Alternatively, *in vivo* labeling techniques relying on localized microinjection of a bolus of concentrated dye can also be used to stain a small subpopulation of a few dozens of cells^34^.

The advent of photoconvertible dyes in the context of biomedical imaging has provided the optics community with a toolkit that allows for the selective labeling of a specific cellular subpopulation of interest where the parameter of interest is identified visually *in situ*. This predictable and convenient conversion process, non-invasively triggered via an external pulsed near-infrared laser source, is further perpetuated to the daughter cells of the converted targets thus greatly facilitating longitudinal studies of heterogeneous cell populations.

The implications for future avenues of research are numerous. Combining this NIR photoconversion methodology with *in vivo* flow cytometry in an animal model^35–37^, for example, would allow one to not only count the number of cells circulating within a particular vascular network, but to identify which cells have already been counted by triggering 750 nm pulsed light irradiation for photoconversion following each detection of a circulating non-converted cell. The DiR labeling the cell would thus be permanently photoconverted and would allow one to obtain highly accurate counts of circulating cells by avoiding repetitious counts.

In another scenario, one may seek to characterize the *in vivo* response to therapy of a growing tumor exposed to injected therapeutics. For instance, animal models fitted with window chambers for direct observation of the implanted tumor may be imaged periodically over a defined time course to study the response of individual cells to the therapy over time^38,39^. By photoconverting responsive (or non-responsive) cells at particular time points in the days and weeks following treatment, a more complete portrait of therapeutic response in the context of cancer treatment can be ascertained.

It is worth noting that the photoconversion of DiR can be completed within seconds rather than minutes of raster-scanning across the targeted area. This can greatly accelerate experimental protocols compared to the use of other photoconvertible agents such as Dendra2 that require conversion times on the order of several minutes in addition to genetic engineering^40,41^. The simplicity of the presented approach, where labeling merely requires a brief incubation period in the presence of the dye without harsh permeabilizing agents, can be easily adopted by scientists with minimal experience using photobiological assays. The potential applications of photoconversion technology in the field of cancer research are thus widely diverse, given the minimally invasive nature and simplicity of the presented methodology.

## Materials and Methods

### Monolayer Cell Culture

The OVCAR5 cell line (Fox Chase Cancer Center) consists of human epithelial carcinoma cells and was derived from the ascitic fluid of a patient with progressive ovarian adenocarcinoma without prior cytotoxic treatment. Cells were plated onto 6-well glass-bottom plates (Cellvis P06-14-0-N, Mountain View, CA) and cultured using Roswell Park Memorial Institute (RPMI) culture medium (Corning 10-040-CV, Corning, NY) supplemented with 10% fetal bovine serum (FBS; Gibco 10437-028, Waltham, MA) and 1% penicillin-streptomycin (Corning 30-001-CI, Corning, NY). Each well was seeded with 6.5 × 10^4^ cells and incubated at 37 °C with 5% atmospheric CO_2_ for 5 days prior to imaging.

In order to label the cells with DiR, the culture medium was first removed from each well and substituted with fresh medium containing 5 *μ*M of DiR. The cells were then incubated for 20 minutes at 37 °C prior to two washing steps with Dulbecco’s phosphate-buffered saline (DPBS without calcium and magnesium; Corning 21-031-CV, Corning, NY). Cells were maintained in DPBS for imaging, and switched back to complete culture medium for longitudinal monitoring.

### 3D Spheroid Culture

Ovarian cancer spheroids were cultured in a manner consistent with previously described methods^29^. Briefly, in order to generate 3D spheroids for *in vitro* culture, 120 *μ*L of Matrigel basement membrane matrix (Corning 356234, Corning, NY) was first dispensed uniformly to fill the glass-bottom well of a 35 mm dish (MatTek Corporation P35G-0-14-C, Ashland, MA). The dish was then incubated at 37 °C for 45 minutes. Next, a suspension of cells in complete medium was prepared with a final concentration of 18,600 cells/mL, such that a total volume of 200 *μ*L of cell suspension was sufficient to seed the gel with roughly 3,700 cells. The volume was dispensed drop-wise onto the gel layer and incubated at 37 °C for another 45 minutes. During the incubation time, a 2% v/v solution of Matrigel dissolved in complete culture medium was prepared, of which 1.8 mL was added to the dish following incubation for a final volume of 2 mL over the gel bed.

Every 2 days following the initial seeding, the culture medium was replaced with fresh medium containing 2% v/v Matrigel and 10 *μ*M DiR in order to ensure complete preliminary staining throughout the entirety of the formed spheroids. By maintaining this routine over the course of 2 weeks, 3D ovarian cancer spheroids with diameters in the few hundred of microns are readily formed^29,30^.

### Spheroid Disaggregation and Fluorescence-Activated Cell Sorting (FACS)

In order to isolate specific cells of interest from a sample of 3D tumor spheroids using FACS, the spheroids first required disaggregation. To this aim, the culture medium was first removed from the glass-bottom dish containing the spheroids. The dish was then filled with 1 mL of dispase solution (Corning 354235, Corning, NY) and incubated at 37 °C for 2 hours to break down the Matrigel matrix. Following incubation, the dish was supplemented with 1 mL of 10 mM EDTA (ethylenediaminetetraacetic acid; Fisher Scientific BP120-500, Waltham, MA) in DPBS to stop the dispase reaction. Next, cells were collected and centrifuged at 250 g for 5 minutes. The supernatant was then removed, and the cell pellet was washed two additional times with fresh DPBS and centrifugation at 250 g for 5 minutes each time. Next, the cell pellet was resuspended in 3 mL of 0.05% trypsin protease solution (GE Healthcare HyClone Trypsin Protease SH30236.01, Chicago, IL) and incubated at 37 °C for 10 minutes. The protease solution was then neutralized by adding 5 mL of complete culture medium following incubation. The cells were then centrifuged one last time at 250 g for 5 minutes, and resuspended in 500 *μ*L of FACS buffer consisting of DPBS supplemented with 5 mL of FBS (1% v/v), 2 mM EDTA, and 25 mM HEPES (4-(2-hydroxyethyl)-1-piperazineethanesulfonic acid; Corning 25-060-CI, Corning, NY). The resulting cell suspension was then sorted using a commercial FACS system (BD FACSAria, Franklin Lakes, NJ), where gating parameters were determined based on the intensities of two fluorescence channels set to capture fluorescence within the 640–680 nm and 750–810 nm ranges.

### Zebrafish Xenograft Model

To generate a short term xenograft model of melanoma, UACC62 melanoma cells were first cultured *in vitro* using complete cell medium (RPMI supplemented with 10% FBS and 1% penicillin-streptomycin). For staining the melanoma cells with DiR, the culture medium was first removed prior to washing with DPBS. Next, the cells were exposed to a 5 *μ*M solution of DiR in complete culture medium and incubated at 37 °C for 20 minutes. The cells were then washed twice with DPBS before reintroducing them to fresh complete culture medium and allowing them to incubate at 37 °C for an additional 20 minutes prior to xenografting.

For the transplantation procedure, zebrafish were handled in full compliance with a protocol approved by the institutional animal care and use committee (IACUC) of Massachusetts General Hospital (MGH IACUC protocol #2011-N-000127). The injection procedure is similar to previously reported methods^42,43^. First, DiR-labeled cells were collected and resuspended such that a final volume of 5 *μ*L containing 1.5 to 2 million cells could be used for the xenograft. Fish were anesthetized by placing them in a dish containing tricaine solution (0.16 mg/mL). Next, each fish was held dorsal side up for the retro-orbital injection, which was performed using a Hamilton syringe. With the bevel side up, the syringe was positioned at an angle of 45° relative to the plane of the fish; if the fish’s eye were a clock, the syringe was placed pointing at the 7:00 position of the eye and inserted 1–2 mm into the tissue. Following the injection, the fish was allowed to recover in fresh water for 7 days prior to photoconversion experiments.

### Fluorescence Microscopy

Confocal fluorescence microscopy was performed using a commercial inverted microscope (Olympus FV1000 IX81, Tokyo, Japan). All image data was recorded with a bit depth of 12, i.e. pixel intensities had a dynamic range from 0 to 4095. The 635 nm continuous wave (CW) laser line was used as the excitation for both standard and photoconverted DiR for all imaging experiments. Lambda-scan image stacks were acquired from 650 nm to 790 nm in 5 nm steps, with a spectral bandwidth of 10 nm at each step.

In the case of the *in vivo* xenograft model, cellular fluorescence was captured sequentially: signals emitted between 650 nm and 690 nm were first recorded, followed by the fluorescence from 760 nm to 800 nm. Z-stacks were acquired over a depth of 300 *μ*m with a step size of 10 *μ*m, using the fish’s eye as a fiducial marker to ensure the same field of view was revisited at each experimental time point.

### Photoconversion

A femtosecond pulsed laser source tuned to a center wavelength of 750 nm (Spectra-Physics InSight DeepSee, Santa Clara, CA) was used to photoconvert the DiR label^19^. The laser light was routed into the optical input port of the confocal microscope described in the previous section. The total power at the output of the microscope objective was kept below 25 mW for all imaging experiments, whether *in vitro* or *in vivo*. The pixel dwell time was limited to 4 *μ*s/pixel, resulting in an approximate scan time of 1 second per frame for a region 512 × 512 pixels in size. Each ROI was thus raster-scanned over the course of 15 to 30 seconds in order to achieve complete and uniform photoconversion of the DiR label across the entire field of view.

### Image Analysis

All image analysis was performed using Matlab R2017a (MathWorks, Natick, MA). In order to generate the fluorescence emission profiles of standard and photoconverted DiR, the pixel intensities of each image slice within a given lambda-scan were summed together. Thus, each spectral image stack was reduced in size from 512 × 512 × 29 pixels to a single 29-element vector, with each element corresponding to the total fluorescence intensity from the entire field of view within a given 10-nm spectral window. A similar process was used to obtain the emission profiles of specific regions of interest within a field of view, where the pixels strictly within the ROI were summed together.

For the xenograft model, given that the z-stacks were acquired sequentially (650–690 nm first, followed by 760–800 nm), image registration was required to properly align the two datasets. To facilitate this process, maximum intensity projections were computed for both z-stacks to generate 2D renditions of the 3D datasets. The projections were then smoothed using a 2D Gaussian kernel with a standard deviation of 0.75. The corresponding fluorescent signals from the 650–690 nm and 760–800 nm channels were then visually matched on a cell-by-cell basis using the control point selection tool in Matlab (*cpselect()*). For each image pair, 150 to 250 control point pairs were manually identified. Next, a geometric transform was computed to fit the control point pairs via a local weighted mean transformation with 50 nearest points using the *fitgeotrans()* Matlab function.

The processed fluorescence intensity images were then overlaid, with the 650–690 nm channel in green (G) and the 760–800 nm channel in red (R), thus generating the images in the top row of Fig. 5. The scatter plots in the middle row were generated by plotting each pixel’s green channel intensity against that of its red channel, resulting in data representation visually reminiscent of flow cytometry scatter plots. Finally, the fluorescent signals were gated using two experimentally-derived criteria to generate the discrete images in the bottom row. First, a pixel was identified as containing DiR if the combined intensity exceeded an empirically determined threshold that is held constant throughout the experimental time course, i.e. if $G+R > Threshold$. Next, a second empirically determined threshold (referred to here as a subthreshold) is set such that if $G > R/3+Subthreshold$, then the DiR was classified as photoconverted. Conversely, if $G < R/3+Subthreshold$, then the DiR was considered standard, i.e. in its native non-converted form. In this manner, pixels that remained black were determined not to contain any detectable levels of DiR. On the other hand, pixels that were either green or red were classified as containing photoconverted or standard DiR, respectively.

## Data Availability

The datasets generated during and/or analyzed during the current study are available from the corresponding author on reasonable request.
